# 

**Published:** 1934

**Authors:** 


### CONTENTS.

| | Page |
|---|---|
| Looking Back. By H. Elwin Harris, M.A., M.B., F.R.C.S. | 1 |
| Vaginal Discharge. By R. S. S. Statham, M.D., Ch.M. | 27 |
| Congenital Word-Blindness. By E. R. Chambers, F.R.C.S. | 41 |
| Nervous Disorders in General Practice. By Frank Bodman, M.B., Ch.B., M.R.C.S., L.R.C.P. | 47 |
| Reviews of Books | 59 |
| Editorial Notes | 70 |
| Obituaries. Dr. Charles Randolph | 71 |
| Edward John Cave, M.D., F.R.C.P. | 73 |
| Meetings of Societies | 73 |
| The Medical Library of the University of Bristol | 75 |
| Publications Received | 78 |
| Local Medical Notes | 79 |

